# Multiple sources of conscious odor integration and propagation in olfactory cortex

**DOI:** 10.3389/fpsyg.2013.00930

**Published:** 2013-12-20

**Authors:** Bernard J. Baars

**Affiliations:** Theoretical Neurobiology, The Neurosciences InstituteLa Jolla, CA, USA

**Keywords:** consciousness, olfaction, global workspace, theoretical neuroscience

## Introduction

How do airborne plumes of molecules docking on olfactory receptors emerge as conscious odors in the brain? How are they interpreted in space, time, and biological meaning? And how do they lead to fast and adaptive decisions and actions?

In general, conscious (reportable) perception supports neural adaptation to novelty, judgments of self-relevance, and voluntary decision-making. Conscious processes have a number of established properties that are markedly different from unconscious ones (Seth et al., 2005). A growing experimental literature has explored conscious perception with a wide array of recording techniques. Similarly, conscious olfaction “as such” can be studied by comparing novel vs. habituated odors, attended vs. unattended ones, and rivaling olfactory percepts, comparable to visual rivalry (Stevenson and Mahmut, 2013). State comparisons of odor processing during waking vs. sleep, general anesthesia, and impaired consciousness are also important.

Neural activity underlying conscious percepts should follow the known psychophysical features of the stimulus. A specific conscious odor should correspond to a specific trajectory in the olfactory perceptual space derived from psychophysical stimulus matching and discrimination (e.g., Berglund et al., 1973; Koulakov et al., 2011).

Global workspace theory (GWT) has been used as a framework for experimental studies of conscious brain processes for more than two decades, leading to a family of related models and experimental predictions. GWT begins by analyzing a reliable set of properties of conscious events (Baars, 1988, 2002). For example, while conscious perception shows limited momentary capacity, it also supports access to non-conscious functions, like memory, executive control, and automatic skills (Baars and Franklin, 2003). By comparison, unconscious stimuli do not afford such very widespread access to unconscious brain capacities.

In general terms, a global workspace (GW) is a functional hub of binding and propagation in a population of loosely coupled signaling elements, such as neurons (Izhikevich, 1999). A GW is commonly compared to the stage of a theater, or a playing field in a large football arena, allowing many specialized knowledge sources to compete and cooperate to resolve focal problems. GW architectures are useful to resolve ambiguous and novel stimuli, such as words in natural language. Conscious percepts often result from a process of ambiguity reduction, and GW architectures have therefore been proposed as models of conscious perception. They are also consistent with highly interactive information flow in the cortico-thalamic (C-T) system (Baars et al., 2013). Edelman et al. (2011) suggest that GW theory is consistent with Neural Darwinism and its many ramifications.

GWT predicted widespread “broadcasting” of conscious events, a prediction that is now widely accepted. In a recent study of visual rivalry in the macaque, content-specific “global broadcasting” from temporal to lateral prefrontal cortex was observed for both oscillatory population signaling and multi-unit recordings (Panagiotaropoulos et al., 2012). Similarly, long-distance cortical phase-linking is associated with the waking state but not slow-wave sleep (see Baars et al., 2013 for a review). In general, conscious sensory input has been repeatedly found to evoke more widespread, high amplitude, and phase-linked oscillations in cortex.

Baars et al. (2013) have proposed that neuronal source coalitions may emerge anywhere in cortex, becoming subjectively conscious and reportable when a convergent winner-take-all source coalition comes to a momentary equilibrium, able to drive many other regions. During the waking state the visual cortex shows reentrant signaling among more than 40 visuotopical maps (Steriade, 2006). In vision the occipito-temporal cortex identifies the perceptual features that emerge in consciousness, from high-resolution visual details to lower-resolution object and event representation (IT and MTL). For the sight of a visual coffee cup or a flower garden, input convergence is believed to occur at high levels of the visual hierarchy, including object perception in area IT and event perception in MTL. However, a simple stimulus, like the sight of a single star on a dark night, might equilibrate early in the visual hierarchy, since areas V1 and LGN have the highest spatial resolution. This highly flexible version of GWT in the C-T system has been called Dynamic GWT (dGWT).

Thus, visual cortex may integrate visual gestalts and broadcast them to frontoparietal, anterior temporal, and subcortical regions. In contrast, non-sensory “feelings of knowing” (FOKs), including expectations and intentions, may arise and propagate from frontal and anterior-temporal regions to caudal sites (Cole et al., 2010; Baars et al., 2013).

Direct brain recordings in human patients show widespread neocortical signaling by way of cross-frequency phase-linking among cortical arrays, especially theta-gamma and alpha-gamma signaling. Single neurons have been shown to phase-adapt to dominant theta oscillations, suggesting a mechanism by which individual neurons may be recruited by population oscillations. Such spatiotemporal coding allows for an extremely rich signaling vocabulary, but specific coding schemes are just beginning to be understood (see Baars et al., 2013 for a review).

## Conceptual framework

Stimulus ambiguity is pervasive in the natural world. Even simple stimuli like a single point of light in a completely dark room are perceived to wander long subjective distances. Ambiguity resolution appears to be a basic feature of sensory systems. For animals the environment is full of unpredictable dangers and opportunities, so that there may be a general evolutionary pressure for brains to resolve focal ambiguities as quickly as possible. Odorant molecules can also be highly ambiguous—they may be sparse, fleeting, masked by other odorants or by attentional distraction, they are often physically degraded, or ambiguous in their biological implications. Conscious olfaction may therefore benefit from the capacity of a GW architecture to concentrate multiple knowledge sources on resolving focal uncertainties.

dGWT suggests that each functional cortical hub passes through two phases, a *convergence* phase combining many cortical sources into single gestalts, and a *broadcasting* phase, in which the gestalt ignites a broadcast of about 100–200 ms, driving widespread adaptation in existing networks. To account for the great range of conscious contents over time, the theory suggests an *open set* of source coalitions, which may broadcast via theta/gamma or alpha/gamma coupling, like AM radio channels competing for a limited band of carrying frequencies (Hoppensteadt and Izhikevich, 1998).

In mammals the C-T core is believed to underlie conscious aspects of perception, thinking, learning, FOKs, felt emotions, visual imagery, working memory, and executive control. The hippocampus and rhinal cortex show similar properties, evolving from pre-mammalian roots. It seems therefore that 3-6-layered cortex may generally support conscious perception (Butler, 2008)^1^.

Because C-T neurons are linked bidirectionally, the system can act as a “unitary oscillatory machine” (Steriade, 2006). The question “which area comes first?” may therefore change dynamically, depending on the balance of expectation-driven vs. stimulus-driven information. Stimulus detection may also change with payoffs: predator odors may have a lower detection threshold than food odors, because the cost of being wrong about predators is higher than the cost of missing food. However, in drought conditions animals may risk greater nearness to predators in order to drink from shared water holes. dGWT allows such context-sensitive factors to shape emergent activity in cortex.

## Multiple neural sources converge to define perceived odors

This paper is focused on conscious odor perception, odor interpretation and odor-guided action control. Since exploratory sniffing is crucial for odor identification, olfaction is directly tied to behavior, including breathing, touching, active tasting, vomeronasal “yawning,” reaching, oral grasping, behavioral stimulus tracking, and the like.

Many olfactory stimuli are reportable “as conscious” with great accuracy by humans, and meet demanding match-to-sample criteria in other animals. Other odorants are processed unconsciously, without direct reportability. The brain differences between conscious and unconscious olfaction are not well understood.

Neuroanatomists traditionally emphasized the differences between six-layered neocortex and ancestral 3–5 layered cortex. Hippocampus is three-layered cortex, but it is often treated as a non-cortical tissue. The entire cortical sheet, including hippocampus and paleocortex, may flexibly support the integration and broadcasting of source coalitions (Baars et al., 2013). For example, olfactory regions project forward to the orbitofrontal cortex. Kay and Sherman (2007) propose that the olfactory bulb may act as an analog of the sensory thalamus. In spite of different gross anatomy, conscious olfaction be similar to conscious vision.

Freeman (2005) has proposed that the microanatomy of dendro-dendritic neuropil provides the basis for such widespread interactivity. Others have focused on the vertical axonal connections of the C-T system (Steriade, 2006). These views are not incompatible, so that cortical signaling may combine both horizontal and vertical signal propagation. Both old and new phylogenetic cortex supports this kind of signaling.

There is debate about the earliest olfactory region where odors are identified as conscious gestalts. Freeman and colleagues suggest that the olfactory bulb analyzes stimuli by destabilizing a pre-existing equilibrium, resulting in a distinctive, high-dimensional attractor landscape incorporating both old and new stimulus parameters. Once the olfactory bulb adapts to the new stimulus, a novel attractor equilibrium propagates as a rapid phase-change in each hemisphere (Kay et al., 1996).

Other approaches point to the posterior piriform and even orbitofrontal cortex for the identification of conscious odors (Gottfried, 2010). Nearby regions are reported to serve multimodal and spatial contextual integration of olfactory stimuli.

From a dGWT perspective, olfactory percepts may come to an equilibrium in different regions of cortex, depending on the precise “source coalition” of active cortical arrays. “Raw” olfactory sensations might arise in the posterior piriform region and spread forward to related regions of cortex. However, “feelings of knowing” about a predictable odor may arise frontally and spread caudally. The peak cortical locus of olfactory percepts may therefore change with expectations, receptor input, the distal spatial and temporal context, selective attention, biological and personal significance, and the current task. In rats, the rate of sniffing (1–12 Hz) also drives theta oscillations, while beta oscillations are reported to encode odor quality (Kay et al., 2009).

## After gestalt formation: centrifugal broadcasting

Global workspace theory suggests both converging source integration and diverging gestalt broadcasting. The best-known example of global propagation in this region is the hippocampal-neocortical dialog, which supports accurate coding of conscious events in episodic memory (Ferkin et al., 2008). The hippocampal complex also seems to support conscious event organization (Baars et al., 2013; Lee and Park, 2013).

Olfactory stimuli have remarkably widespread effects in the brain, including the limbic system, hypothalamus, amygdala, reward pathway, orbitofrontal, and insular cortex (Savic, 2005). One example of long-distance brain signaling may be Kay and Freeman's (1998) reported widespread cortical gamma coherence in the rat. According to these authors, coherent oscillatory “wave packets” may broadcast odor gestalts to other regions, using theta-to-beta phase-linked oscillations. In mammals, prefrontal regions support voluntary decisions evoked by conscious stimuli. The ability to report a conscious stimulus, and to perform match-to-sample tasks, plausibly requires prefrontal cortex (Asplund et al., 2010).

Olfaction is sometimes thought to be “primitive,” but it is actually highly sophisticated. In nature, odors must be identified, interpreted, and acted on, to determine how long ago a tree was scent-marked, and whether the marking animal was a predator or a potential mate. The ancestral smell brain has been proposed to be an autonomous olfactory-motor system, since odors trigger rapid, adaptive behavior, as in detecting and running from the smell of a predator. Many factors, like wind direction, ambient temperatures, mating readiness, health and immune status, inferred age of the stimulus, concentration gradients, and the nature of the scent-marked surface must be interpreted accurately. Intervening rain or snow changes the composition of odor mixtures, which must still be identified correctly. Accurate olfaction and taste are also vital for food sampling and toxin avoidance, digestion, elimination, reproduction and sexual behavior, immunity, biological cycles, the health/illness dimension and its emotions, and the vagus-insula interoceptive system.

## Conclusion

Conscious perception emerges from a complex, highly interactive process of resolving ambiguous and context-dependent stimuli. Baars et al. (2013) propose that sensory cortex gives rise to neuronal source coalitions that emerge into reportable consciousness when they drive many other cortical regions. By contrast, non-sensory cortex may give rise to FOKs, such as the “tip of the tongue” experience. Animals following an odorant trail often show a kind of expectant “tip of the nose” pose, tasting the air, and the ground for more information about a suspected odor source.

Because odorants may be fleeting, sparse, intermittent, masked, physically degraded, or ambiguous, conscious odor perception may also benefit from the interactivity of a GW capacity. In mammals both the new and old cortex support such interactivity.

The GW framework has been used to study conscious vision and audition. It may also help to clarify a world of conscious odors.

## Footnotes

^1^Crick and Koch (2005) have suggested that cortico-claustral circuits may be involved in consciousness, given the widespread input and output connections of the claustrum. However, the exact role of the claustrum is still unclear (Smythies et al., 2012).
